# Altered Lipid Metabolism in Recovered SARS Patients Twelve Years after Infection

**DOI:** 10.1038/s41598-017-09536-z

**Published:** 2017-08-22

**Authors:** Qi Wu, Lina Zhou, Xin Sun, Zhongfang Yan, Chunxiu Hu, Junping Wu, Long Xu, Xue Li, Huiling Liu, Peiyuan Yin, Kuan Li, Jieyu Zhao, Yanli Li, Xiaolin Wang, Yu Li, Qiuyang Zhang, Guowang Xu, Huaiyong Chen

**Affiliations:** 1 Key Research Laboratory for Infectious Disease Prevention for State Administration of Traditional Chinese Medicine, Tianjin Institute of Respiratory Diseases, Haihe Clinical College of Tianjin Medical University, Tianjin, China; 2 CAS Key Laboratory of Separation Sciences for Analytical Chemistry, Dalian Institute of Chemical Physics, Chinese Academy of Sciences, Dalian, 116023 China; 3 Department of Respiratory Medicine, Tianjin Haihe Hospital, Tianjin, China; 4 Department of Nutrition, Tianjin Haihe Hospital, Tianjin, China; 5 Department of Basic Medicine, Tianjin Haihe Hospital, Tianjin, China; 6 Department of Gastroenterology, Tianjin Haihe Hospital, Tianjin, China

## Abstract

Severe acute respiratory syndrome-coronavirus (SARS-CoV) and SARS-like coronavirus are a potential threat to global health. However, reviews of the long-term effects of clinical treatments in SARS patients are lacking. Here a total of 25 recovered SARS patients were recruited 12 years after infection. Clinical questionnaire responses and examination findings indicated that the patients had experienced various diseases, including lung susceptibility to infections, tumors, cardiovascular disorders, and abnormal glucose metabolism. As compared to healthy controls, metabolomic analyses identified significant differences in the serum metabolomes of SARS survivors. The most significant metabolic disruptions were the comprehensive increase of phosphatidylinositol and lysophospha tidylinositol levels in recovered SARS patients, which coincided with the effect of methylprednisolone administration investigated further in the steroid treated non-SARS patients with severe pneumonia. These results suggested that high-dose pulses of methylprednisolone might cause long-term systemic damage associated with serum metabolic alterations. The present study provided information for an improved understanding of coronavirus-associated pathologies, which might permit further optimization of clinical treatments.

## Introduction

Between 2002 and 2003, severe acute respiratory syndrome (SARS) caused by coronavirus (SARS-CoV) affected over 30 countries on five continents. Soon after, HCoV-NL63 was identified in a seven-month-old infant with bronchiolitis and conjunctivitis in 2004^1^, and followed by HCoV-HKU1 in 2005^2^. Later, the Middle East Respiratory Syndrome coronavirus (MERS-CoV) rapidly spread worldwide with fatality rates up to 30%^3^. Several reports have recently demonstrated the existence of a SARS-like coronavirus, which indicates that coronavirus-associated respiratory tract infections continue to have a significant epidemic potential^4–6^. Therefore, there remains an urgent requirement for standardization of treatments in order to develop more effective interventional strategies for coronavirus-associated diseases.

Clinical treatments implemented following the sudden outbreak of SARS-CoV included empirical and experimental types, although their effectiveness and side effects remain controversial. Antiviral drugs, including ribavirin, lopinavir, and ritonavir, were prescribed soon after SARS-CoV was identified as the causative agent^7, 8^. Type I interferon was also used owing to its inhibitory effect on SARS-CoV replication^9^. Furthermore, the use of convalescent plasma or immunoglobulin was reported for the treatment of SARS^10^. More frequently, corticosteroids, including methylprednisolone alone or in combination with antiviral drugs, were administered to SARS patients to prevent immunopathological lung damage^11^, although the timing and dosage regimens for steroid administration were controversial^12, 13^. The administration of high-dose pulses of methylprednisolone has been associated with psychic derangements, acute myopathy, and osteonecrosis^14–16^. Considering the fact that these disorders usually progress to a chronic state, we propose that the SARS survivors may suffer from metabolic disturbances, resulting in systemic pathologies in the long-term. Metabolites, including phosphatidylinositol (PI) and lysophosphatidylinositol (LPI), are associated with cellular entry and/or egress of respiratory viruses^17, 18^. However, it remains unknown whether or not serum levels of PI and LPI are disregulated during recovery of SARS infections.

In the present study, metabolomics based on ultra-high-performance liquid chromatography-mass spectrometry (UPLC-MS) and gas chromatography-mass spectrometry (GC-MS) was adopted to assess the comprehensive metabolite profile of serum or urine samples from SARS patients 12 years following their clinical recovery. It was observed that the recovered SARS patients were affected by various serum metabolic disorders predominantly associated with lipid metabolism, including hyperlipidemia (HL), cardiovascular abnormality (CVA), and abnormal glucose metabolism (AGM).

## Results

### Demographics of recovered SARS patients at 12 years following recovery

Of the 31 recruited SARS-CoV survivors, 25 ultimately completed all study procedures, while 6 discontinued participation in the study (three dropouts, one protocol deviation, and two missing comparable healthy controls). The 25 patients that completed the study included 5 men and 20 women, with an average age of 47 years, an average body weight of 67 kg, and an average body mass index of 24 kg/m^2^, which were not significantly different from healthy volunteers (Table 1). None of the recovered SARS patients had smoking or drinking habits. During SARS-CoV infection, these patients had been treated with methylprednisolone at an average of 5,454 mg for a mean period of 36 days, with a maximum daily dose of 381 mg (Table 1).

**Table 1:** General participant characteristics (means ± SD).

	Recovered SARS patients (n = 25)	Healthy volunteers (n = 25)	*P*
Male/female (n)	5/20	5/20	
Age (years)	47 ± 12	47 ± 11	0.486
Body weight (kg)	67 ± 13	63 ± 12	0.107
BMI (kg/m^2^)	24 ± 3	24 ± 4	0.230
Smoking	0	8%	
Alcohol drinking	0	8%	
Methylprednisolone (mg)	5454 ± 3153	0	
Duration of treatment (days)	36 ± 7	0	
Max. daily pulse (mg)	381 ± 306	0	
Hospitalization since recovery (%)	48	12 (8% deliveries)	
Lung infection since recovery (%)	64	12	
Osteonecrosis (%)	60	0	
Hyperlipidemia (%)	68	40	
Cardiovascular abnormality (%)	44	0	
Abnormal glucose metabolism (%)	60	16	
Red blood cells in urine (%)	33	4	
Urine protein (%)	8	0	
Below normal range (%)			
Neutrophil ratio	20	12	
Triiodothyronine	8	0	
Above normal range (%)			
TGs	44	16	
VLDL-C	16	0	

SARS, severe acute respiratory syndrome; BMI, body mass index; TGs, triglycerides; VLDL-C, very low-density lipoprotein concentration.

**Figure 1:**
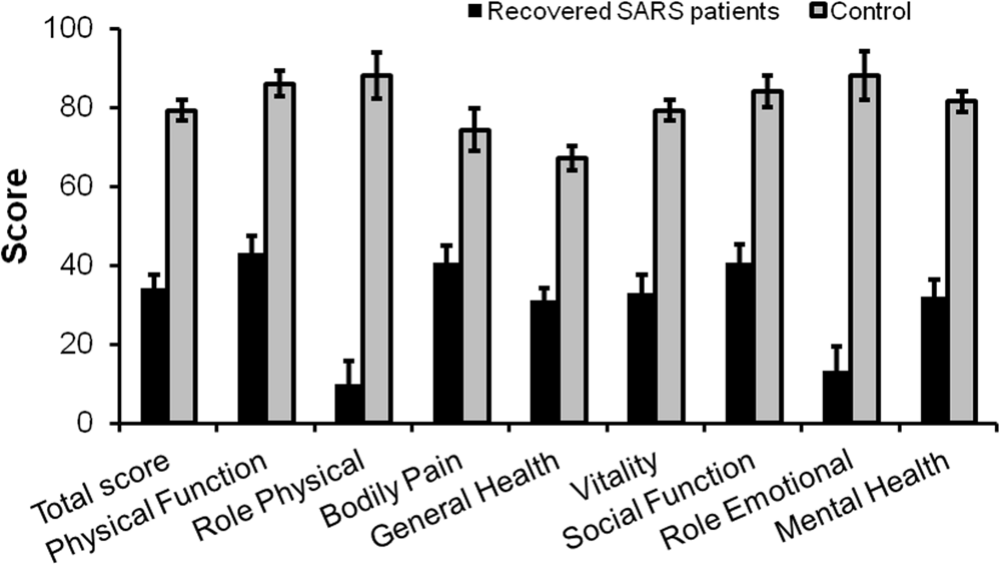
MOS 36-item Short Form Health Survey (SF-36) results for severe recovered respiratory syndrome patients 12 years after recovery.

The responses to the questionnaires indicated that, since recovery, 12 of the 25 recovered SARS patients (48%) had been readmitted to hospital at least once: four had experienced osteonecrosis of the femoral head; one due to fetal death; and two had been diagnosed with hysteromyoma, one with osphyarthrosis, one with cervical spondylosis, one with a spinal cord tumor, and one with breast cancer (data not shown). Sixteen of the recovered SARS patients (64%) had suffered lung infections during the past 12 years (Table 1). The susceptibility of respiratory tract was also suggested by the fact that 13 of the 25 subjects (52%) reported having had a cold at least five times in the past year, as compared with the healthy volunteers, in which all patients reported having had less than five colds in the past year (data not shown). In addition, the recovered SARS patients had a tendency to have HL (68%), CVA (44%), and AGM (60%) (Table 1). Urine screening detected red blood cells in 8 of the 24 SARS subjects (33%); of these, 2 patients (8%) exhibited abnormal urine protein levels. More subjects in the recovered SARS group had a neutrophil ratio and triiodothyronine levels below the normal ranges (20% and 8%, respectively) than in the healthy volunteers (12% and 0%, respectively). Similarly, as compared with the healthy volunteers, more subjects in the recovered SARS group had triglyceride (TG) levels and very low-density lipoprotein concentrations (VLDL-C) above the normal ranges (Table 1 and S1). Together with the SF-36 Health Survey (Fig. 1), these data demonstrated that the recovered SARS patients had a poor quality of life 12 years following recovery, and were susceptible to inflammation, tumors, and glucose and lipid metabolic disorders.

### Altered serum metabolomes

**Figure 2:**
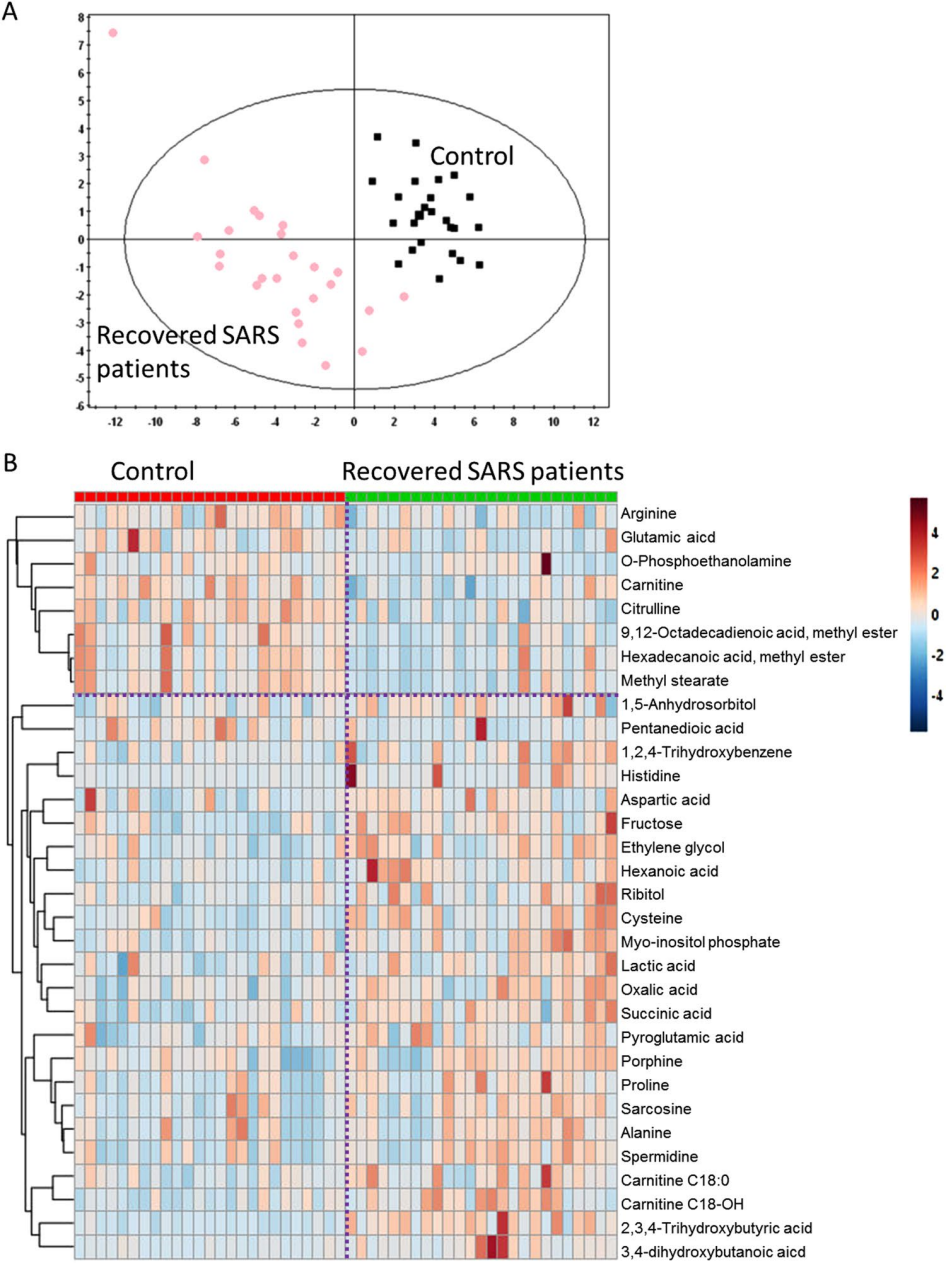
Differential serum metabolic profiles between recovered SARS and healthy volunteers. (**A**) OPLS-DA score plot separating the recovered SARS and healthy volunteers (Control). (**B**) Heat map of significantly altered metabolites detected by gas chromatography-mass spectrometry and liquid chromatography-mass spectrometry.

Although no significant differences were observed in the abundance of metabolites in the urine samples of the SARS and healthy volunteers, partial least squares-discriminant analysis (PLS-DA) showed significant differences in the serum metabolome between recovered SARS patients and healthy controls (Fig. 2A). These data points were clustered into two distinct groups in the plot map, obviously separating the recovered SARS patients and healthy controls (Fig. 2A). The majority of the differentially expressed metabolites, including 2,3,4-trihydroxybutyric acid, cysteine, long-chain acylcarnitine (carnitines C18:0 and C18-OH), fructose, 1,2,4-trihydroxybenzene, pyroglutamic acid, and myo-inositol phosphate, were upregulated in the serum samples from recovered SARS patients compared with the healthy volunteers (Fig. 2B). Metabolites that were decreased in the recovered SARS patients compared with healthy volunteers included methyl esters such as 9,12-octadecadienoic acid and hexadecanoic acid, free carnitine, and arginine (Fig. 2B).

**Figure 3:**
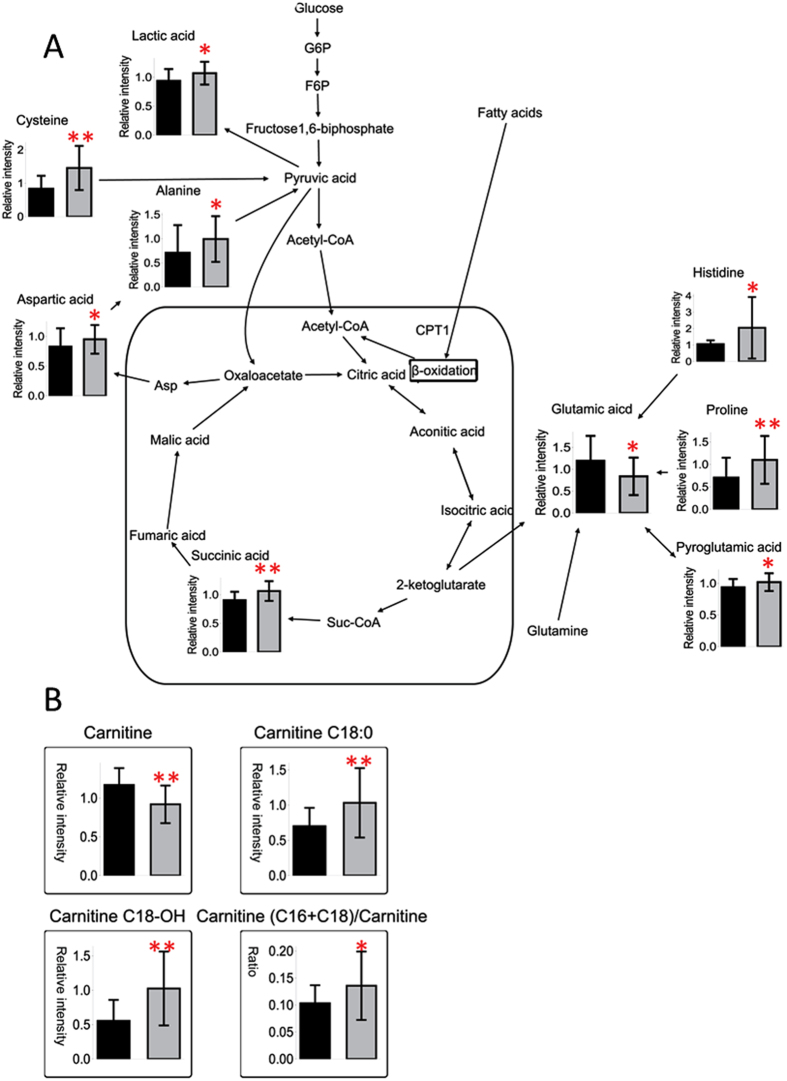
Mapping of differential metabolites related to energy metabolism (**A**). Relative serum levels of differentially expressed free carnitine, acylcarnitines with long chains, and the ratio of carnitines (C16 + C18) to carnitine (**B**). Data shown as mean ± SEM. * < 0.05, ** < 0.01.

**Figure 4:**
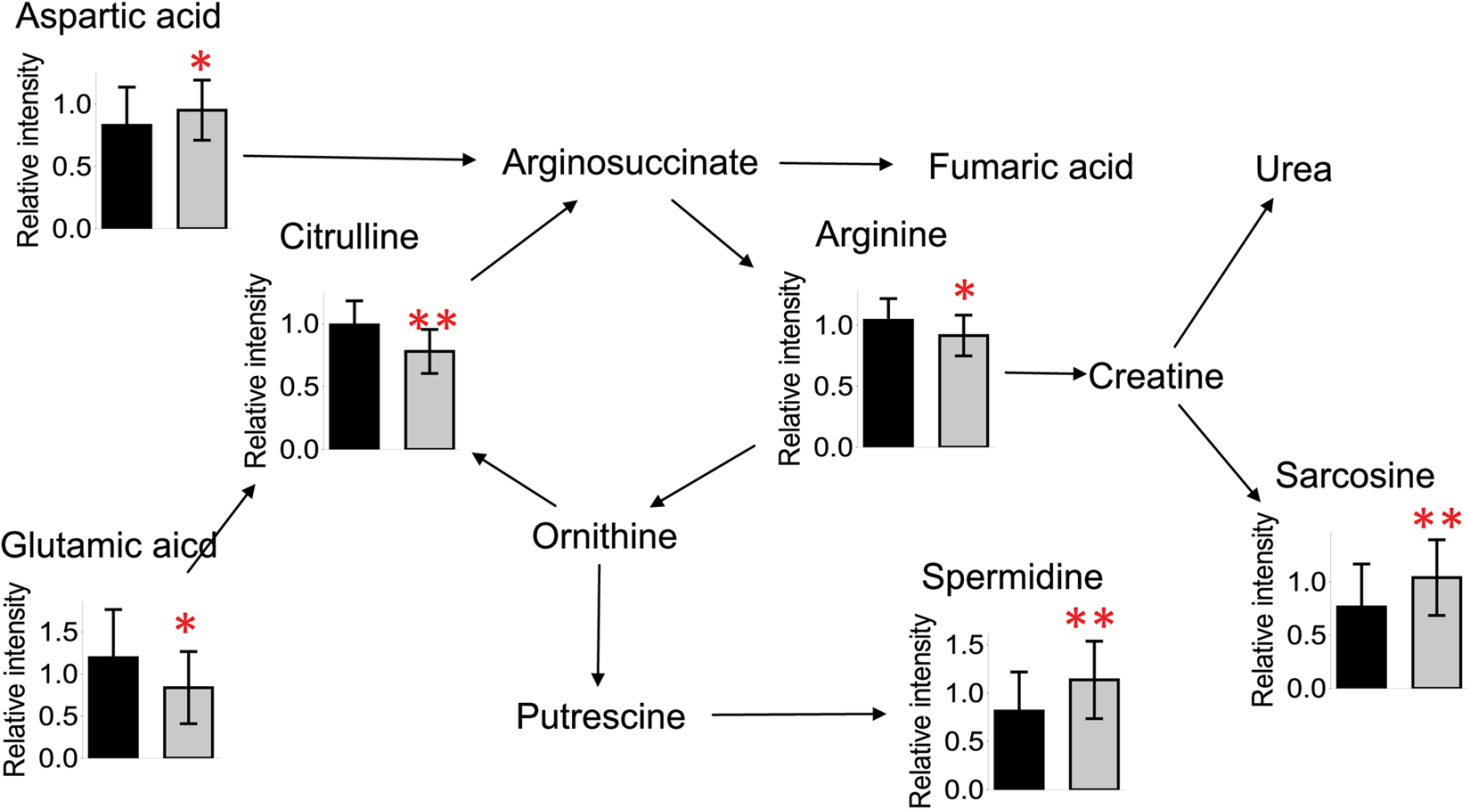
Mapping of differential metabolites related to arginine and proline metabolism. Data shown as mean ± SEM. * < 0.05, ** < 0.01.

To investigate the potential metabolic disruptions in recovered SARS patients, the alterations in metabolite abundance were further associated with metabolic pathways. The levels of lactic acid derived from glycolysis were elevated in recovered SARS patients compared with healthy volunteers (Fig. 3). Similarly, the levels of cysteine, aspartic acid, and alanine, which can be converted to pyruvic acid, as well as the levels of succinic acid, which is an intermediate of the tricarboxylic acid cycle (TCA) cycle, were elevated in the recovered SARS patients compared with healthy volunteers. In addition, the serum levels of proline, histine, and pyroglutamic acid, which can be converted into glutamic acid and enter the TCA cycle for further energy production or substance synthesis, were increased in the SARS patients compared with healthy volunteers. While the levels of free carnitine were decreased in the SARS patients compared with healthy volunteers, the levels of carnitines C18:0 and C18-OH were increased (Fig. 3). The ratio of carnitine (C16 + C18) to free carnitine ((C16 + C18)/C0) was also increased (Fig. 3), which suggested that fatty acids could easily enter the mitochondria for further β-oxidation in the recovered SARS patients. The serum levels of sarcosine and spermidine were increased in recovered SARS patients compared with healthy volunteers, whereas the levels of citrulline and arginine, which are intermediates of the urea cycle, were decreased (Fig. 4), thus suggesting that there was an increased requirement for arginine in the urea cycle in the recovered SARS patients.

### Disturbed lipid metabolism

The elevated serum lipids included free fatty acids (FFAs 10:0, 15:0, 17:0, 17:1, 20:4, 22:1, and 22:2, accounting for 6.4% of all detected FFAs), lysophosphatidylcholines (LPCs 18:2, 20:2, 21:3, and 22:6, accounting for 23.7% of all detected LPCs), lysophosphatidylethanolamines (LPEs 18:1 and 22:6, accounting for 19.4% of all detected LPEs), and phosphatidylglycerol (PG 36:0) (Table 2). Of note, there were comprehensive elevations of LPIs and PIs in recovered SARS patients. Among 3 LPIs and 14 PIs examined, LPIs 18:0, 18:2, and 20:4 (accounting for 100% of all detected LPIs) and PIs 34:1, 34:2, 36:1, 36:2, 36:3, 36:4, 38:3, 38:4, 38:5, 38:6, 40:5, and 40:6 (accounting for 95.7% of all detected PIs) were increased (Table 2). The lipids that were decreased in recovered SARS patients compared with the healthy volunteers included long-chain FFAs (FFAs 25:0 and 26:0, accounting for 0.026% of all detected FFAs), ether phosphatidylethanolamines (PE O-s 37:4, 38:6, and 40:7), PG 36:2, phosphatidylserines (PSs 38:4 and 40:6, accounting for 3.8% of all detected PSs), sphingomyelins (SMs 34:1;3, 36:0;2, 37:4;2, and 38:0;2, accounting for 1.9% of all detected SMs), and TGs (TGs 44:2, 44:3, and 47:0, accounting for 0.69% of all detected TGs).

**Table 2:** Differential lipids in serum from recovered SARS patients versus Healthy volunteers.

Lipids	Ratio	*P*	Lipids	Ratio	*P*	Lipids	Ratio	*P*
FFA 10:0	1.14	0.039	LPE 22:6	1.27	0.041	PI 38:4	1.61	<0.001
FFA 15:0	1.11	0.008	LPI 18:0	2.13	<0.001	PI 38:5	1.47	0.001
FFA 17:0	1.08	0.032	LPI 18:2	1.75	<0.001	PI 38:6	1.55	0.001
FFA 17:1	1.10	0.005	LPI 20:4	1.74	<0.001	PI 40:5	1.46	0.001
FFA 20:4	1.29	0.025	PE O-37:4	0.63	<0.001	PI 40:6	1.36	0.001
FFA 22:1	1.29	0.001	PE O-38:6	0.61	0.017	PS 38:4	0.85	0.010
FFA 22:2	1.15	0.037	PE O-40:7	0.50	0.001	PS 40:6	0.78	0.002
FFA 25:0	0.86	0.029	PG 36:0	1.38	0.002	SM 34:1;3	0.86	0.020
FFA 26:0	0.90	0.006	PG 36:2	0.58	0.003	SM 36:0;2	0.62	<0.001
FFA (odd)	1.09	0.019	PI 34:1	1.49	<0.001	SM 37:4;2	0.61	<0.001
FFA (odd)/FFA(even)	1.08	0.023	PI 34:2	1.78	<0.001	SM 38:0;2	0.60	<0.001
LPC 18:2	1.18	0.026	PI 36:1	1.64	<0.001	TG 44:2	0.61	<0.001
LPC 20:2	1.28	0.008	PI 36:2	1.57	<0.001	TG 44:3	0.64	<0.001
LPC 21:3	1.06	0.003	PI 36:3	1.39	0.002	TG 47:0	0.85	0.019
LPC 22:6	1.30	0.021	PI 36:4	1.76	0.002	total SM/total (SM + PC)	0.92	0.019
LPE 18:1	1.26	0.035	PI 38:3	1.53	0.001			

FFA, free fatty acids; FFA(odd), fatty acids with odd number carbon chains; FFA (odd)/FFA(even), ratio of fatty acids with odd number carbon chains to fatty acids with even number carbon chains; LPC, lysophosphatidylcholine; LPE, lysophosphatidylethanolamine; LPI, lysophosphatidylinositol; PE O-, ether phosphatidylethanolamine; PG, phosphatidylglycerol; PI, phosphatidylinositol; PS, phosphatidylserine; SM, sphingomyelin; TG, triglyceride; total SM/total (SM + PC), ratio of total SM to the sum of total SM and total phosphatidylcholine.

**Figure 5:**
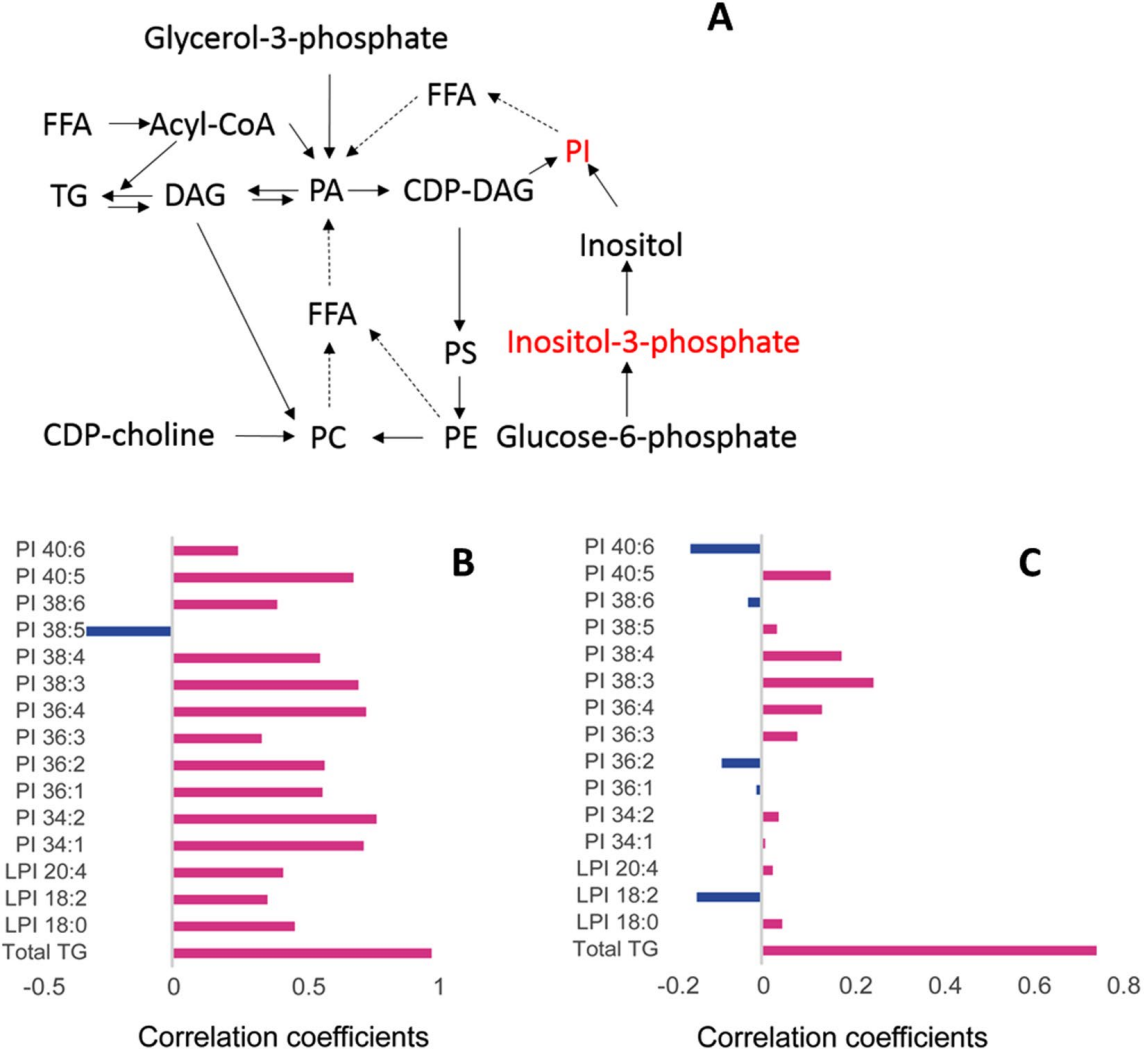
Major pathways of phospholipid and neutral lipid synthesis. (**A**) Dashed lines show fatty acid rearrangement through the remodeling process. Correlation analysis of very low-density lipoprotein, phosphatidylinositol, lysophosphatidylinositol, and total triglycerides in (**B**) recovered SARS and (**C**) healthy volunteers (Control).

Several pathways exist for the synthesis of phospholipids and neutral lipids, including fatty acid rearrangement via a remodeling process (Fig. 5A). Serum levels of inositol-3-phosphate and PIs were significantly increased in recovered SARS patients compared with the healthy volunteers (Fig. 5A). Additionally, the PI levels were positively correlated with the levels of very low-density lipoprotein (VLDL) in recovered SARS patients (Fig. 5B and C). Furthermore, VLDL was positively correlated with total TG in both the control and recovered SARS groups (Fig. 5B and C). These results suggested that de novo synthesis of PIs was promoted during or after SARS-CoV infection.

**Figure 6:**
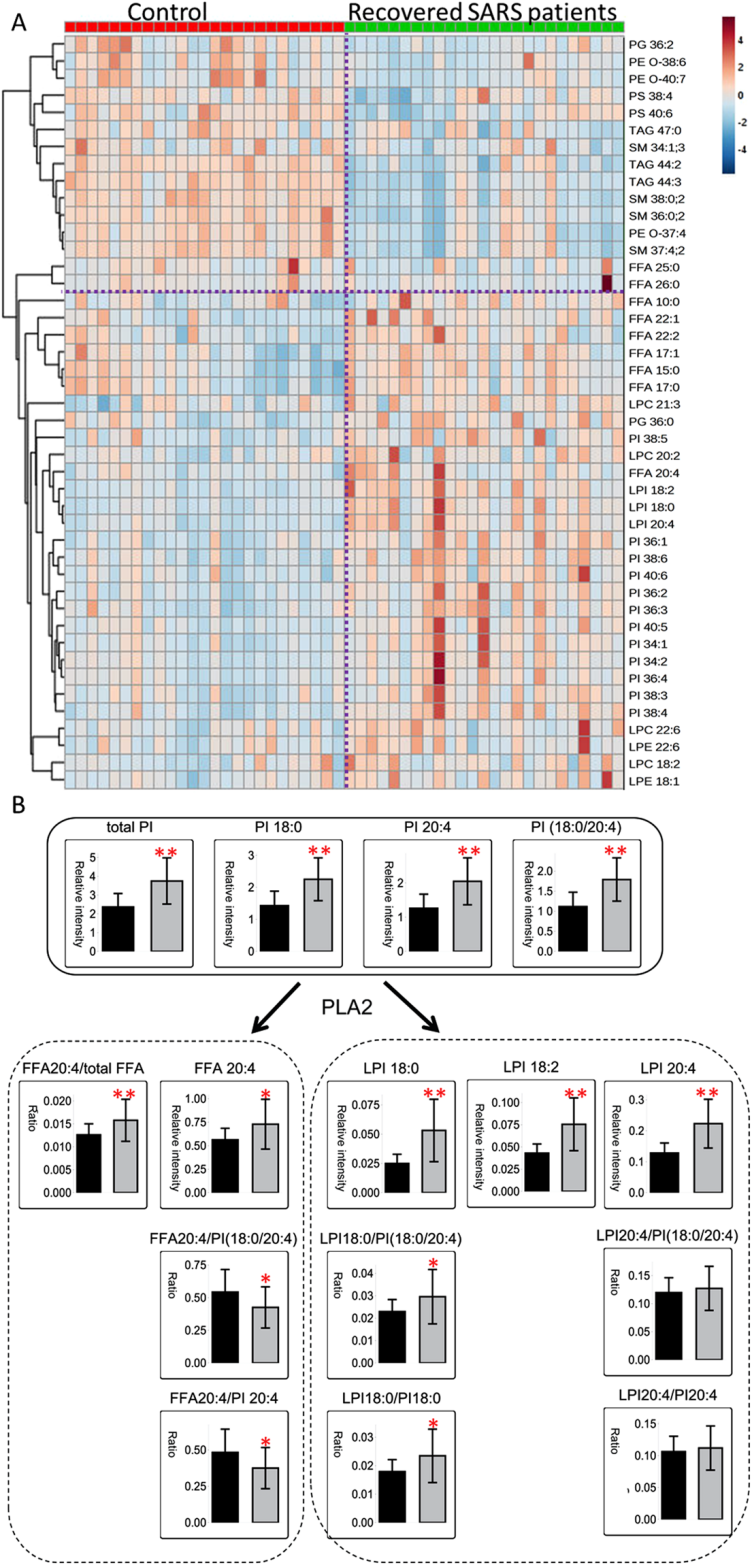
(**A**) Cluster analysis of differentially expressed lipids. (**B**) Fatty acids as a result of phosphatidylinositol remodeling in recovered SARS patients sensitive to phospholipase A2.

A cluster analysis was performed to further assess the associations among the differentially expressed lipids. In recovered SARS patients, the three LPIs (LPIs 18:0, 18:2, and 20:4) and FFA 20:4 were most closely clustered, followed by the PIs and LPC 22:6, LPE 22:6, LPC 18:2, and LPE 18:1 (Fig. 6A). Products of phospholipase A2 (PLA2), including FFA 20:4, LPIs, LPCs, and LPEs, showed strong positive correlations (Fig. 6A). LPI 18:0, LPI 18:2, and LPI 20:4 were associated with their respective precursor PIs 18:0, 18:2, and 20:4 (cij = 0.68, 0.6, and 0.67, respectively). Nevertheless, FFA 20:4 was correlated with LPI 18:0/PIs (18:0/20:4) and LPI 18:0/PI 18:0 (Cij = 0.72 and 0.74, respectively), further illustrating the enhanced levels of PI remodeling in recovered SARS patients. More LPIs were produced, which was associated with the release of FFA 20:4, in recovered SARS patients compared with healthy volunteers, which may have been due to the elevated levels of PIs; thus, SARS patients may be more sensitive to PLA2 activity (Fig. 6B). The LPI 18:0/PI 18:0 ratio was elevated, while the FFA 20:4/PI 20:4 ratio was significantly decreased, in recovered SARS patients, which indicated that more LPIs remained following PLA2 cleavage, whereas the released FFA 20:4 from PIs was rapidly used for eicosanoid synthesis (Fig. 6B).

### Effect of steroid treatment on lipid metabolism

High-dose steroid treatment could contribute to the alterations of lipid metabolism in serum of recovered SARS patients. Next we investigated the short-term effects of methylprednisolone treatment on non-SARS patients with severe pneumonia. Twenty three non-SARS patients with severe pneumonia treated with steroid included 13 men and 10 women (67 ± 14 years old, 301 ± 235 mg of methylprednisolone for a mean period of 9 days). Twenty three non-SARS patients with severe pneumonia without steroid treatment included 15 men and 8 women (68 ± 8 years old). In coincidence with the former findings, the levels of detected PIs were significantly higher in steroid-treated group than those without steroid treatment (Table 3). Although the doses of steroid used for these non-SARS patients were much less than those for SRAS patients, these results, to some extent, suggested that steroid treatment could increase levels of serum PIs.

**Table 3:** Differential phosphatidylinositols in serum from steroid-treated non-SARS patients with severe pneumonia versus non-SARS patients with severe pneumonia without steroid treatment.

Lipids	steroid-treated patients vs. non-SARS patients with severe pneumonia	
	Ratio	*P*
PI 32:1	3.16	<0.001
PI 34:1	1.83	<0.001
PI 34:2	1.54	<0.001
PI 35:2	1.55	<0.001
PI 36:1	1.73	0.003
PI 36:2	1.37	0.012
PI 36:3	1.57	0.013
PI 36:4	1.77	<0.001
PI 37:4	1.66	<0.001
PI 38:3	1.49	0.021
PI 38:4	1.42	0.013
PI 38:5	1.60	0.001
PI 38:6	1.34	0.022
PI 40:4	1.40	0.001

## Discussion

Although SARS-CoV survivors benefited from clinical treatments that targeted the virus and inflammation, the recovered patients in the current study continued to experience a poor quality of life 12 years after their infections. Our previous study reported that the pulmonary ventilation function of recovered SARS survivors was normal^19^. A significant proportion of these recovered patients were reported as having lung infections, HL, CVA, AGM, or other sequelae. Serum metabolomics analyses identified systemic metabolic alterations in these patients. The predominant metabolic dysregulations were observed in the serum, with serum levels of PIs, LPIs, FFAs, and LPCs showing the greatest disruptions. Compared with the other differentially expressed metabolites, LPIs and PIs were markedly upregulated in the recovered SARS patients. The serum levels of LPIs and PIs were not influenced by sequelae observed in these survivors (Tables S2–S5). Subjects were regrouped based on metabolic syndromes. Statistical analyses of such subgroups indicated that the identified metabolic alterations were not associated with HL, CVA, or AGM (Figs S1–S3). But, our results suggested that early steroid treatment could have an effect on the serum levels of PIs or LPIs in recovered SARS patients. However, in rat experiments, the levels of the detected PIs in rats intraperitoneally injected with methylprednisolone groups (10 or 30 mg/kg) were comparable to those of the untreated rats (Table S6), suggesting that the effect of steroid treatment on serum lipid metabolism is more complex than what we thought.

PIs have previously been related to viral infections associated with lungs. For example, the membrane binding and penetration of the Ebola virus fusion peptide requires the presence of Pl in the target membrane^17^. Downstream elements of the PI signaling pathway have been shown to be involved in viral entry. The Ebola virus matrix protein, VP40, was recently shown to require Pl 4,5-bisphosphate for extensive oligomerization and viral egress^18^. Pl 4-kinase IIIβ (PI4KB) is hypothesized to have a role in the cellular entry of pseudoviruses bearing the SARS-CoV spike protein, and cell entry mediated by the SARS-CoV spike protein was strongly inhibited by knockdown of PI4KB^20^. Therefore, upregulated PIs in SARS patients may promote SARS-CoV infection and entry. However, Pls were also shown to suppress respiratory syncytial virus infection in mice^21^.

LPIs are thought to have a critical role in glucose homeostasis^22^. A large proportion of the recovered SARS patients reported glucose metabolic disorders, including hyperinsulinemia, insulin resistance, hyperglycemia, and type 1 or 2 diabetes, a few years prior to this study. LPI stimulates insulin release^23^, and activation of the G-protein coupled receptor 55 (GPR55) can increase plasma insulin levels and glucose tolerance^24^. Lipidomic analyses also demonstrated increasing LPI levels in a pre-type 1 diabetic mouse model^25^. In the present study, increased rates of glycolysis with few effects on the TCA cycle were observed in the recovered SARS patients, suggesting an association between LPI and glucose metabolism in recovered SARS patients. As a result of the broad expression of GPR55 across various tissues, LPI exerts diverse regulatory roles, including in the excitability of dorsal root ganglion neurons^26^, intestinal inflammation^27^, and the migration and orientation of human breast cancer^28^. This may explain the various symptoms observed in the recovered SARS patients. LPI-associated disorders may persist as long as the high serum LPI levels remain.

There were a number of limitations associated with the present study. First, we cannot rule out the possibility that some of the patients with glucose metabolic disorders had taken medication prior to the analyses, which may have influenced their physiological status. Second, although we endeavored to recruit as many recovered patients as possible, the sample size was small. Third, the best controls for the investigation of steroid treatment effects were to recruit SARS patients without steroid treatment. Because of lack of those controls, non-SARS patients with severe pneumonia with or without steroid treatment were collected in present study, but they may not exactly mimic the two key elements of SARS infection: steroid treatment and SARS virus infection. Therefore, the reasons for the marked increases in serum levels of LPIs and PIs in recovered SARS patients require further investigation. Changes in metabolite levels observed in our study may not reflect the real situation exactly.

As the primary organ damaged by SARS infection, lung function was improved gradually over years. The profound metabolic disruptions may also be directly or indirectly related to initial damages in lungs, which were resulted from SARS-CoV infection. Or the lung damage may be resulted from complex organism response to infection and high-dose pulse of steroid treatment. More importantly, these findings may improve the understanding of coronavirus-induced pathological mechanisms, and may aid in the development of improved measures for the treatment and control of coronavirus infections that continue to threaten human health worldwide.

## Materials and Methods

### Ethics Statement

Written informed consent was obtained from all study participants. The institutional review board of Tianjin Haihe Hospital provided written approval and judged that all methods in the study met relevant ethical guidelines and regulations (ethical # 2013HHKT-01).

### Subjects

Thirty-one SARS survivors of the 2002–2003 outbreak, as well as 31 age-matched, consenting healthy volunteers who were medical staff at the Tianjin Haihe Hospital, were recruited in 2014. General participant information, including age, disease duration, and medication, was collected using a standard form. Student’s t-test (two-tailed) was used to evaluate the differences between the two groups. Furthermore, each participant completed a symptom questionnaire regarding his or her medical history since SARS infection. After fasting overnight, serum and urine samples were collected in the morning from all patients for routine clinical measurements and metabolomics analysis.

For further lipid metabolism investigation, serum samples were also collected from 46 non-SARS patients with severe pneumonia, half of these patients were treated with methylprednisolone.

### Clinical measurements

Evaluation of the general physical ability of the patients included quality of life scoring (Medical Outcomes Study Short Form-36, SF-36), Harris Hip score, the six-min walk test, assessment of pulmonary ventilation function, and the bronchial dilation test. Routine clinical chemical analyses were conducted using fasting blood samples at the Tianjin Haihe Hospital.

### LC-MS-based serum metabolic profiling

Serum samples were prepared as described before^29^. Briefly, protein precipitation was performed on the serum samples. Aliquots of 200 μL were drawn for each sample, and four volumes of acetonitrile (with internal standard mixture of carnitine C2:0-d3, carnitine C10:0-d3, carnitine C16:0-d3, LPC 19:0, free fatty acid (FFA) 16:0-d3, FFA 18:0-d3, chenodeoxycholic acid-d4, cholic acid-d4, leu-d3, Val-d8, tryptophan-d5, phenylalanine-d5 and leucine-enkephalin) were added. After thorough vortex and centrifugation (13,000 × g for 10 min at 4 °C), two aliquots of 300 μL supernatant were pipetted for drying in CentriVap Centrifugal Vacuum Concentrators (Labconco, MO). The dried residues were stored at −80 °C until analysis. LC-MS-based metabolic profiling was performed on an ultra-high-performance liquid chromatograph (Waters, USA) coupled to a tripleTOF™ 5600 plus mass spectrometer (Applied Biosystems, Foster City, CA) equipped with an electrospray source. The dried serum samples were dissolved in acetonitrile (ACN)/water (1:4 v/v) and, following centrifugation at 4 °C, 5 μL supernatant was loaded. For the positive mode, a BEH C8 (100 mm × 2.1 mm × 1.7 μm; Waters, Milford, MA) column was used. The gradient was initiated with 10% B mobile phase (0.1% formic acid in ACN), which was maintained for 1 min, followed by linearly increasing to 40% B within 4 min, and then to 100% B at 17 min, which was maintained for 5 min. The mobile phase was rapidly changed to 90% A (0.1% formic acid) within 0.1 min, and the total run time for each injection was 25 min, including a post-equilibration of 2.9 min.

### GC-MS-based serum metabolic profiling

The derivatization procedure and data acquisition were performed as described previously, with some modifications^29^. Briefly, the temperature for both oximation and silylation reactions was changed to 37 °C and the oximation reaction time was reduced to 1.5 h. Serum metabolic profiling was performed in a pseudotargeted mode using a GCMS-QP 2010 Plus quadrupole mass analyzer coupled to an AOC-20i autosampler (Shimadzu, Kyoto, Japan). Chromatographic separation was performed on a DB-5 MS capillary column (30 m × 250 μm × 0.25 μm; J&W Scientific, Folsom, CA). First, the quality control (QC) sample was analyzed in full scan mode with a mass-to-charge ratio (m/z) scan range of 33–600 Dalton to obtain the target ion for selected ion monitoring (SIM) scanning. The parameters for the full scan were as follows: the flow rate of the carrier gas (helium) was constant at 1.2 mL/min; the oven temperature began at 70 °C, which was maintained for 3 min, followed by a linear increase to 310 °C at 5 °C/min, and finally holding for 6 min at 310 °C; the temperature of the inlet and interface was 280 °C, and that of the ion source was 240 °C; the detector voltage was set at 1.05 kV; the solvent delay was 5.1 min; and the event time was initiated at 0.5 s. With the integrated SIM scan table, batch analysis for all samples was conducted in pseudotargeted mode with all MS parameter settings the same as those used in full scan mode, but with the event time adjusted to 0.2 s.

### Lipidomic analysis of serum samples

For lipidomics analysis, 30-µL serum aliquots were pipetted for each sample and 200 µL methanol containing 8 lipid internal standards (PC38:0, PE34:0, LPC19:0, SM12:0, triglyceride 45:0 (TG45:0), Cer17:0, FFA16-d3 and FFA18-d3) was added, followed by a 30-sec vortex. Then, 400 µL chloroform was added and the mixture was vortexed for another 30 seconds. After that, 120 µL water was added and the extraction mixture, vortexed, and centrifuged (8,000 x g for 10 min at 4 °C) to form aqueous-organic separation phases. Two, 200 µL aliquots of the upper organic phase were drawn and dried, respectively, in a vacuum centrifuge and stored at −80 °C until analysis. Quality control (QC) samples were produced by pooling equal serum aliquots from each sample, containing the average metabolome information from the real samples included in this study. For LC-MS, GC-MS metabolic profiling and lipidomics, QC sample pretreatment was performed using the same method as that used for the actual patient samples. The QC sample was run every 10 real samples to evaluate the repeatability of sample pretreatment and for batch analysis of each analytical platform. The lipidome was analyzed using UPLC (Waters) and triple-quadrupole time-of-flight mass spectrometry (AB Sciex) systems. Prior to the analysis, the dried serum samples were reconstituted in 20 µL CHCl_3_-MeOH solution (2:1 v/v) and then diluted in a mixed solvent containing ACN-MeOH-H_2_O (65:30:5 v/v/v). Subsequently, 5 µL of the diluted samples were separated using a C8 ACQUITY^TM^ column (150 × 2.1 mm, 1.7 µm internal diameter). The column temperature and elution rate were set at 55 °C and 0.26 mL/min, respectively. The mobile phases A and B, which were ACN:H_2_O (6:4 v/v) and isopropanol: ACN (9:1 v/v), respectively, both contained 10 mM ammonium acetate. The initial gradient started at 32% B, which was maintained for 1.5 min, followed by a linear increase to 85% B in 14 min, and then to 97% B within 0.1 min. This condition was maintained for 2.4 min at 97% B, followed by a decline to 32% B within 0.1 min. The total run time was 20 min, including a post-equilibration time of 1.9 min. The mass spectrometry signal was scanned from a m/z of 400–1500 Dalton in both positive and negative ion modes. The capillary voltages were set at 4.0 kV and −3.8 kV for positive and negative modes, respectively. The capillary temperature was 350 °C.

### Data preprocessing for serum GC-MS, LC-MS, and lipidomics analysis

For the GC-MS analysis, unique ions for metabolites in the final SIM scan table were imported to GC-MS solution software for batch integration. For the LC-MS analysis, raw data were first imported to Peak-view software for automated peak detection, alignment, and integration. After checking the derived peak table, known metabolites that had been missed or had a relative standard deviation of greater than 30% during the automated peak picking were extracted and integrated manually. Further unknown ions detected in blank samples were deleted if their intensity proportion in the blank sample was more than 1% compared with the average intensity levels in the experimental samples. The 80% rule was applied to remove peaks with missing values surpassing 20% in both groups. For mass spectrometry response normalization in both GC-MS and LC-MS analyses, the raw peak areas for each ion or metabolite were normalized to that of adjacent QCs, with the local linear regression of QC intensities selected. For the lipidomics analysis, peak picking was performed using Lipid-View software (AB Sciex). Owing to their complexity, the lipid compounds were detected by extracting the ion chromatograms to avoid the “black box” associated with automatically pretreated data. Adjustment of the integration parameters was performed in certain cases. The peak areas of each detected lipid compound were normalized to those of the spiked lipid internal standards according to the different lipid classes.

### Statistical analyses

Mann-Whitney U tests and hierarchical cluster analyses were performed using the Multi Experiment Viewer. The significance level for the univariate analysis was set at p < 0.05. Corresponding *q* values for each significant metabolite were calculated to evaluate false discovery rates in multiple significant tests. An overall safer bootstrap method was used for estimation with the step 0.05 set for lambda. For robust *q* value calculation from small *p* values with finite samples, the “use robust method” was further selected. The critical value for *q* was set as 0.2. The column plots were drawn using Vanted, with metabolic pathways based on Kyoto Encyclopedia of Genes and Genomes (KEGG) classifications^30^.

## Electronic supplementary material

Supplemental data
