# Supplementary material for: Altered Lipid Metabolism in Recovered SARS Patients Twelve Years after Infection
Source: Sci Rep. 2017 Aug 22;7:9110. doi: 10.1038/s41598-017-09536-z (PMC5567209; doi:10.1038/s41598-017-09536-z)
Supplement: Supplementary file 1 — Supplemental data [file 41598_2017_9536_MOESM1_ESM.doc]

**Altered Lipid Metabolism in Recovered SARS Patients Twelve Years after Infection**

Qi Wu^1,#^, Lina Zhou^2,#^, Xin Sun^3,#^, Zhongfang Yan^4,#^, Chunxiu Hu^2^, Junping Wu^3^, Long Xu^3^, Xue Li^5^, Huiling Liu^6^, Peiyuan Yin^2^, Kuan Li^5^, Jieyu Zhao^2^, Yanli Li^2^, Xiaolin Wang^2^, Yu Li^5^, Qiuyang Zhang^5^, Guowang Xu^2^,*, Huaiyong Chen^1,5^,*

^1^Key Research Laboratory for Infectious Disease Prevention for State Administration of Traditional Chinese Medicine, Tianjin Institute of Respiratory Diseases, Haihe Clinical College of Tianjin Medical University, Tianjin, China.

^2^Key Laboratory of Separation Science for Analytical Chemistry, Dalian Institute of Chemical Physics, Chinese Academy of Sciences, Dalian 116023, China.

^3^Department of Respiratory, Tianjin Haihe Hospital, Tianjin, China.

^4^Department of Nutrition, Tianjin Haihe Hospital, Tianjin, China.

^5^Department of Basic Medicine, Tianjin Haihe Hospital, Tianjin, China.

^6^Department of Gastroenterology, Tianjin Haihe Hospital, Tianjin, China.

**^#^**These authors contributed equally to this work.

**Supporting information**

**Reagents**

HPLC-grade acetonitrile, methanol, isopropanol (Merck, Darmstadt, Germany), formic acid, ammonium bicarbonate, ammonium acetate (Sigma- Aldrich, St. Louis, MO, USA), and laboratory-prepared Milli-Q water (Millipore, Bedford, MA), were used to prepare the mobile phases. Additional chloroform (Sigma- Aldrich, St. Louis, MO, USA) was used during serum pretreatment for lipidomics analysis. Chemical standards used as internal standards and for compound characterization were purchased from Sigma-Aldrich (St. Louis, MO, USA). Methylprednisolone was purchased from Pfizer Manufacturing Belgium NV.

**Animals and treatments**

Rat experiments were performed according to the protocol approved by Tianjin Haihe Hospital Animal Care and Use Committee (protocol # 2015HHLL04) and in direct accordance with Ministry of Science and Technology of the People's Republic of China on Animal Care guidelines. All surgeries were performed under anesthesia and all efforts were made to minimize suffering.

Sprague Dawley (SD) rats aged 12 weeks were purchased from Huafukang Biotech (Beijing), and were randomly divided into three groups (n=5/group): the untreated group and intramuscularly injected with 10 or 30 mg/kg methylprednisolone sodium succinate groups. After 6 weeks of treatment, the rats were sacrificed and tail vein blood was harvested for metabolomics analysis.

**Figure S1**. Differential metabolites or lipids influenced by hyperlipidemia (HL) in female SARS survivors. Data shown as mean ± SEM. *: <0.05, **: <0.01.

**
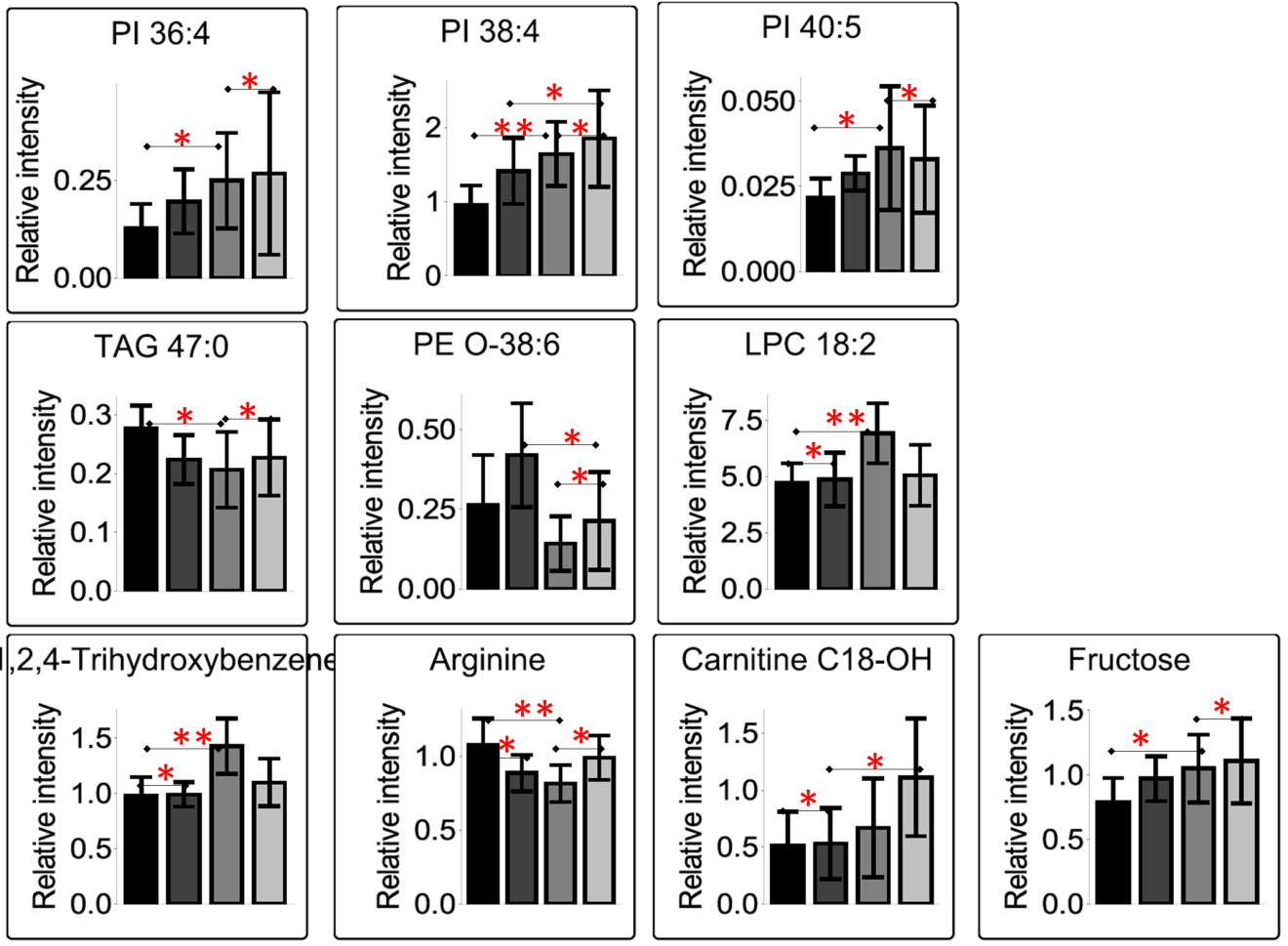
**

**Figure S2.** Percentage distributions of subjects with or without cardiovascular abnormalities (CVAs) in recovered SARS and control groups (A); Differential metabolites or lipids influenced by CVAs (B). Data shown as mean ± SEM. *: <0.05, **: <0.01.

**
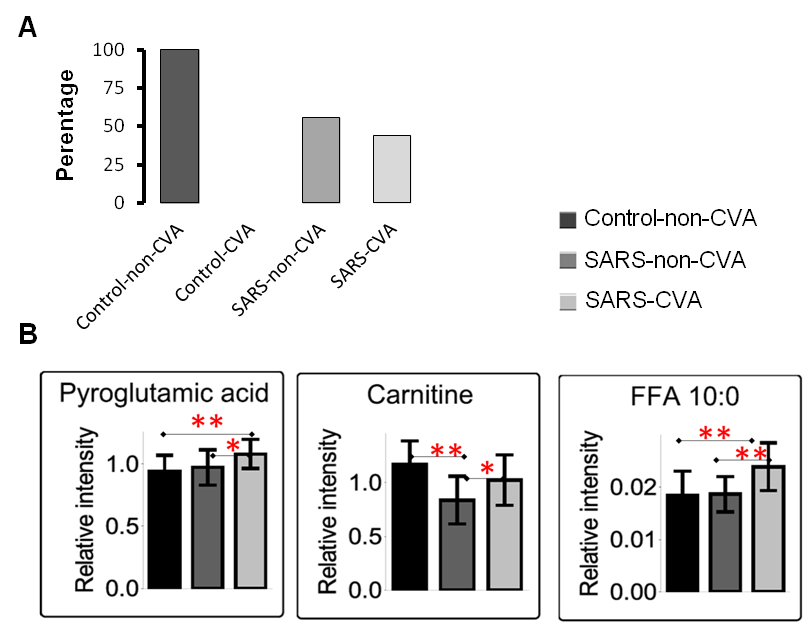
**

**Figure S3.** Percentage distributions of subjects with or without abnormal glucose metabolism (AGM) in recovered SARS and control groups (A); Differential metabolites or lipids influenced by AGM (B). Data shown as mean ± SEM. *: <0.05, **: <0.01.

**
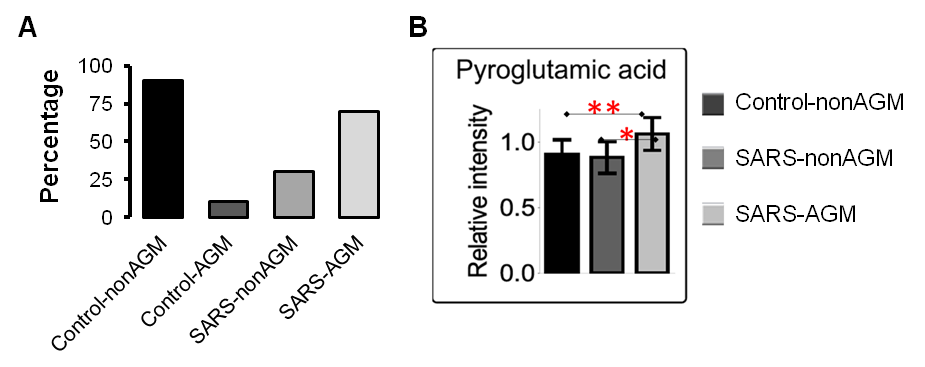
**

**Table S1. Clinical serum analysis (means ± SD).**

|  | **SARS** | **Control** | ***p*** | Normal range |
| --- | --- | --- | --- | --- |
| Thrombin time (seconds) | 15.1 ± 1.2 | 14.2 ± 0.9 | 0.007 | 16-24 |
| platelet distribution width (PDW) | 13.5 ± 2.6 | 12.2 ± 1.6 | 0.065 | 9-17 |
| Thyroxine (nmol/L) | 120.7 ± 28.9 | 96.0 ± 14.5 | <0.001 | 66-181 |
| Triiodothyronine (nmol/L) | 1.99 ± 0.36 | 1.64 ± 0.22 | <0.001 | 1.3-3.1 |
| PCO_2_ (mmHg) | 36.8 ± 2.3 | 38.1 ± 2.5 | 0.033 | 35-45 |
| Bicarbonate radical | 23.6 ± 1.2 | 24.4 ± 1.7 | 0.063 | 21.4-27.3 |
| Cl^-^ (mmol/L) | 107.0 ± 1.7 | 107.9 ± 1.8 | 0.121 | 98-106 |
| Anion gap (AG) (mmol/L) | 10.3 ± 1.8 | 8.7 ± 3.2 | 0.003 | 8-16 |
| Neuron-specific enolase (ng/ml) | 10.3 ± 3.5 | 3.2 ± 1.8 | <0.001 | 0-15.2 |
| Total protein (g/L) | 75.3 ± 4.3 | 73.7 ± 3.9 | 0.130 | 60-88 |
| Albumin (g/L) | 47.5 ± 2.8 | 46.8 ± 2.2 | 0.357 | 35-52 |
| The white ball ratio | 1.75 ± 0.30 | 1.77 ± 0.26 | 0.641 | 1.50-2.50 |
| Total bilirubin (μmol/L) | 10.3 ± 4.5 | 10.8 ± 3.6 | 0.560 | 1.71-21.9 |
| Bilirubin direct (μmol/L) | 3.9 ± 1.5 | 3.8 ± 1.0 | 0.627 | 0-6.8 |
| Alanine aminotransferase (U/L) | 23.2 ± 21.0 | 18.9 ± 17.3 | 0.502 | 0-40 |
| Aspartate aminotransferase (U/L) | 23.0 ± 16.6 | 17.6 ± 6.7 | 0.254 | 0-40 |
| r-glutamyltransferase (U/L) | 29.5 ± 33.3 | 24.4 ± 25.5 | 0.992 | 8-64 |
| Total bile acid (μmol/L) | 4.1 ± 2.7 | 3.3 ± 2.0 | 0.314 | 0-12 |
| Choline esterase (U/L) | 8721 ± 1369 | 8017 ± 2153 | 0.218 | 5400-13200 |
| Urea nitrogen (mmol/L) | 4.6 ± 1.2 | 4.5 ± 1.0 | 0.662 | 2.9-8.2 |
| Creatinine (μmol/L) | 61.9 ± 14.0 | 61.8 ± 10.3 | 0.808 | 35-106 |
| Uric acid (μmol/L) | 259 ± 56 | 271 ± 73 | 0.677 | 137-488 |
| Organophosphorus (mmol/L) | 1.14 ± 0.14 | 1.17 ± 0.13 | 0.491 | 0.8-1.5 |
| Glycated hemoglobin (%) | 5.3 ± 0.5 | 5.4 ± 0.5 | 0.711 | 4.8-5.9 |
| Fasting serum glucose (mmol/L) | 5.38 ± 0.72 | 5.51 ± 0.75 | 0.641 | 3.89-6.11 |
| Lactate dehydrogenase (U/L) | 168.8 ± 34.2 | 162.8 ± 25.6 | 0.485 | 114-240 |
| Adenosine deaminase (U/L) | 9.3 ± 2.4 | 8.4 ± 1.8 | 0.186 | 0-20.0 |
| TG (mmol/L) | 1.86 ± 1.70 | 1.08 ± 0.55 | 0.073 | 0.4-1.51 |
| TC (mmol/L) | 5.00 ± 1.08 | 5.13 ± 1.25 | 0.727 | 2.33-5.17 |
| HDL-C (mmol/L) | 1.51 ± 0.41 | 1.67 ± 0.39 | 0.221 | 1.04-1.74 |
| LDL-C (mmol/L) | 3.10 ± 0.90 | 3.34 ± 1.09 | 0.467 | 0-3.12 |
| VLDL-C (mmol/L) | 0.39 ± 0.52 | 0.13 ± 0.13 | 0.030 | 0-0.78 |

TC, total cholesterol; HDL-C, high density lipoprotein concentration; LDL-C, low density lipoprotein concentration.

**Table S2.** Phosphatidylinositol (PI) and lysophophoinositol (LPI) ratios in recovered SARS patients compared to control both without hyperlipidemia (HL).

| **Lipids** | **SARS/Control** | ***P*** |
| --- | --- | --- |
| LPI 18:0 | 2.58 | 0.002 |
| LPI 18:2 | 2.29 | <0.001 |
| LPI 20:4 | 1.97 | 0.001 |
| PI 34:1 | 1.71 | 0.039 |
| PI 34:2 | 2.35 | 0.008 |
| PI 36:1 | 1.68 | 0.013 |
| PI 36:2 | 1.87 | 0.003 |
| PI 36:3 | 1.56 | 0.039 |
| PI 36:4 | 1.95 | 0.017 |
| PI 38:3 | 1.67 | 0.026 |
| PI 38:4 | 1.71 | 0.002 |
| PI 38:5 | 1.61 | 0.070 |
| PI 38:6 | 1.4 | 0.138 |
| PI 40:5 | 1.67 | 0.013 |
| PI 40:6 | 1.15 | 0.187 |

When calculating q values for the significance tests for PIs or LPIs, lamda was set to 0, with other parameters set as described in the method part. The calculated *q* values for significant PIs or LPIs were smaller than 0.11.

**Table S3.** Phosphatidylinositols (PIs) and lysophosphatidylinositols (LPIs) in recovered SARS patients compared to controls both without abnormal glucose metabolism (AGM).

| **Lipids** | **SARS/Control** | ***P*** |
| --- | --- | --- |
| LPI 18:0 | 2.65 | 0.008 |
| LPI 18:2 | 1.98 | 0.009 |
| LPI 20:4 | 1.91 | 0.014 |
| PI 34:1 | 1.81 | 0.020 |
| PI 34:2 | 2.07 | 0.045 |
| PI 36:1 | 1.79 | 0.023 |
| PI 36:2 | 1.66 | 0.020 |
| PI 36:3 | 1.38 | 0.162 |
| PI 36:4 | 2.05 | 0.032 |
| PI 38:3 | 1.57 | 0.028 |
| PI 38:4 | 1.67 | 0.002 |
| PI 38:5 | 1.52 | 0.023 |
| PI 38:6 | 1.62 | 0.023 |
| PI 40:5 | 1.62 | 0.028 |
| PI 40:6 | 1.4 | 0.008 |

When calculating q values for the significance tests for PIs or LPIs, lamda was set to 0, with other parameters set as described in the method part. The calculated *q* values for significant PIs or LPIs were smaller than 0.11.

**Table S4.** Phosphatidylinositol (PI) and lysophosphatidylinositols (LPI) ratios in recovered SARS patients compared to control both without cardiovascular abnormality (CVA).

| **Lipids** | **SARS/Control** | ***P*** |
| --- | --- | --- |
| LPI 18:0 | 2.18 | <0.001 |
| LPI 18:2 | 1.86 | <0.001 |
| LPI 20:4 | 1.8 | <0.001 |
| PI 34:1 | 1.51 | 0.004 |
| PI 34:2 | 1.92 | 0.005 |
| PI 36:1 | 1.67 | <0.001 |
| PI 36:2 | 1.66 | <0.001 |
| PI 36:3 | 1.36 | 0.014 |
| PI 36:4 | 1.79 | 0.018 |
| PI 38:3 | 1.56 | 0.007 |
| PI 38:4 | 1.66 | <0.001 |
| PI 38:5 | 1.37 | 0.018 |
| PI 38:6 | 1.53 | 0.009 |
| PI 40:5 | 1.55 | 0.004 |
| PI 40:6 | 1.37 | 0.030 |

When calculating q values for the significance tests for PIs or LPIs, lamda was set to 0, with other parameters set as described in the method part. The calculated *q* values for significant PIs or LPIs were smaller than 0.11.

**Table S5.** Phosphatidylinositols (PIs) and lysophosphatidylinositols (LPIs) in recovered SARS patients compared to control patients both with normal thyroid function.

| **Lipids** | **SARS/Control** | ***P*** |
| --- | --- | --- |
| LPI 18:0 | 2.09 | <0.001 |
| LPI 18:2 | 1.78 | <0.001 |
| LPI 20:4 | 1.74 | <0.001 |
| PI 34:1 | 1.48 | <0.001 |
| PI 34:2 | 1.81 | <0.001 |
| PI 36:1 | 1.58 | <0.001 |
| PI 36:2 | 1.56 | <0.001 |
| PI 36:3 | 1.38 | 0.003 |
| PI 36:4 | 1.71 | 0.009 |
| PI 38:3 | 1.54 | 0.002 |
| PI 38:4 | 1.55 | <0.001 |
| PI 38:5 | 1.4 | 0.004 |
| PI 38:6 | 1.49 | 0.003 |
| PI 40:5 | 1.41 | 0.007 |
| PI 40:6 | 1.27 | 0.007 |

When calculating q values for the significance tests for PIs or LPIs, lamda was set to 0, with other parameters set as described in the method part. The calculated *q* values for significant PIs or LPIs were smaller than 0.11.

**Table S6.** Effect of methylprednisolone treatment on PIs detected in rats treated with low (L)-dose (10 mg/kg) or high (H)-dose (30 mg/kg) of methylprednisolone sodium succinate respectively.

|  | **H/Control** | ***P*** | **L/Control** | ***P*** |
| --- | --- | --- | --- | --- |
| PI(16:0/16:1) | 1.87 | 0.354 | 0.88 | 0.462 |
| PI (16:0/18:1) | 1.47 | 0.171 | 1.04 | 0.568 |
| PI (16:0/18:2) | 0.83 | 0.171 | 0.80 | 0.142 |
| PI (16:0/20:3) | 1.70 | 0.354 | 1.12 | 0.744 |
| PI (16:0/20:4) | 0.83 | 0.171 | 0.87 | 0.165 |
| PI (16:0/22:4) | 1.49 | 0.453 | 1.15 | 0.462 |
| PI (16:0/22:5) | 1.10 | 0.895 | 0.99 | 0.870 |
| PI (16:0/22:6) | 0.77 | 0.145 | 0.70 | 0.050 |
| PI (17:0/18:2) | 1.14 | 0.508 | 0.88 | 0.462 |
| PI (17:0/20:4) | 1.25 | 0.270 | 1.25 | 0.462 |
| PI (18:0/16:1) | 1.28 | 0.402 | 1.02 | 0.624 |
| PI (18:0/18:1) | 0.94 | 0.402 | 0.70 | 0.028 |
| PI (18:0/18:2) | 66.58 | 0.566 | 12.63 | 0.806 |
| PI (18:0/18:3) | 1.83 | 0.627 | 0.98 | 0.624 |
| PI (18:0/20:3) | 1.43 | 0.171 | 1.34 | 0.102 |
| PI (18:0/20:4) | 0.88 | 0.566 | 1 | 0.870 |
| PI (18:0/20:5) | 1.22 | 0.965 | 1.30 | 0.568 |
| PI (18:0/22:5) | 1.04 | 0.691 | 1.11 | 0.414 |
| PI (18:0/22:6) | 0.85 | 0.508 | 0.90 | 0.414 |
| PI (18:1/18:1) | 0.99 | 0.757 | 0.92 | 0.462 |
| PI (18:1/18:2) | 1.53 | 0.102 | 0.73 | 0.121 |
| PI (18:1/20:4) | 1.07 | 0.965 | 1 | 0.744 |
| PI (19:0/20:4) | 0.73 | 0.825 | 0.71 | 0.683 |

PI, phosphatidylinositol.
